# 4-Allyl-2-meth­oxy­phenyl 3,4-dichloro­benzene­sulfonate

**DOI:** 10.1107/S1600536811044163

**Published:** 2011-10-29

**Authors:** Ya-Tuan Ma, Zhao-Feng Gao, Qi-Chao Liu, Gang Jin, Jin-Ming Gao

**Affiliations:** a College of Science and College of Life Sciences, Northwest A&F University, Yangling Shaanxi 712100, People’s Republic of China; b College of Science, Northwest A&F University, Yangling Shaanxi 712100, People’s Republic of China

## Abstract

The title compound, C_16_H_14_Cl_2_O_4_S, was obtained by the reaction of eugenol (4-allyl-2-meth­oxy­phenol) and 3,4-dichloro­benzene­sulfonyl chloride. The dihedral angle between the benzene rings in the mol­ecule is 40.53 (4)°. No significantly short inter­molecular contacts are observed in the crystal structure.

## Related literature

For the synthesis of eugenol derivatives, see: Sadeghian *et al.* (2008 ▶). For a related structure, see: Ma *et al.* (2010 ▶).
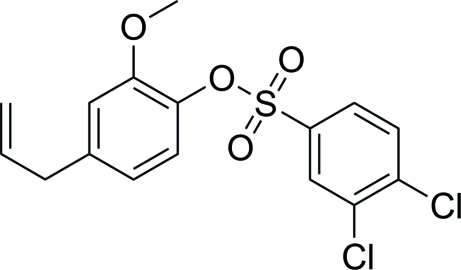


## Experimental

### 

#### Crystal data

C_16_H_14_Cl_2_O_4_S
*M* *_r_* = 373.23
Triclinic, 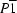

*a* = 8.8694 (8) Å
*b* = 9.7501 (9) Å
*c* = 10.3796 (11) Å
α = 83.369 (2)°
β = 76.196 (1)°
γ = 80.038 (1)°
*V* = 855.95 (14) Å^3^
*Z* = 2
Mo *K*α radiation
μ = 0.52 mm^−1^
*T* = 298 K
0.45 × 0.40 × 0.30 mm

#### Data collection

Bruker SMART APEX CCD area-detector diffractometer
Absorption correction: multi-scan (*SADABS*; Sheldrick, 1996 ▶) *T*_min_ = 0.801, *T*_max_ = 0.860
4290 measured reflections
2967 independent reflections
1762 reflections with *I* > 2σ(*I*)
*R*_int_ = 0.017

#### Refinement

*R*[*F* ^2^ > 2σ(*F* ^2^)] = 0.048
*wR*(*F* ^2^) = 0.140
*S* = 1.02
2967 reflections
209 parameters
H-atom parameters constrained
Δρ_max_ = 0.36 e Å^−3^
Δρ_min_ = −0.33 e Å^−3^

### 

Data collection: *SMART* (Bruker, 2001 ▶); cell refinement: *SAINT* (Bruker, 2001 ▶); data reduction: *SAINT*; program(s) used to solve structure: *SHELXS97* (Sheldrick, 2008 ▶); program(s) used to refine structure: *SHELXL97* (Sheldrick, 2008 ▶); molecular graphics: *SHELXTL* (Sheldrick, 2008 ▶); software used to prepare material for publication: *SHELXTL*.

## Supplementary Material

Crystal structure: contains datablock(s) I, global. DOI: 10.1107/S1600536811044163/bh2390sup1.cif

Structure factors: contains datablock(s) I. DOI: 10.1107/S1600536811044163/bh2390Isup2.hkl

Supplementary material file. DOI: 10.1107/S1600536811044163/bh2390Isup3.cml

Additional supplementary materials:  crystallographic information; 3D view; checkCIF report

## Figures and Tables

**Fig. 1.**
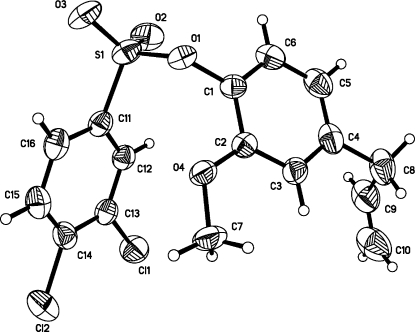
The molecular structure of the title molecule. Displacement ellipsoids are drawn at the 30% probability level.

**Fig. 2.**
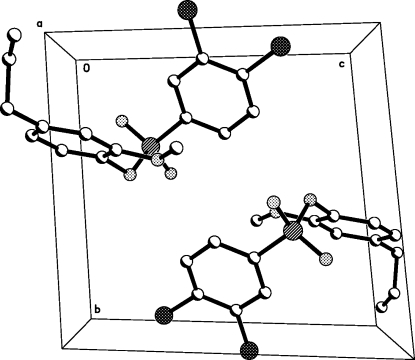
Packing diagram.

## supplementary crystallographic information

### Comment

In this paper, we present the structure of the title compound (Fig. 1), which was synthesized by the reaction of eugenol and 3,4-dichlorobenzenesulfonyl chloride (Sadeghian *et al.*, 2008). We previously reported a compound of this type (Ma *et al.*, 2010). In the molecular structure, the bond lengths and angles are normal and the dihedral angle between the aromatic rings is 40.53 (4)°. The crystal packing exhibits no significantly short intermolecular contacts.

### Experimental

492 mg of eugenol (3 mmol), triethylamine (4 mmol), 3,4-dichlorobenzenesulfonyl chloride (3 mmol), and 40 ml of dichloromethane were mixed in a 100 ml flask. After 2 h under stirring at 278 K, the crude product was obtained. The crystals were obtained by recrystallization from methanol.

### Refinement

The positions of all H atoms were fixed geometrically and C—H bond lengths fixed to 0.93 (aromatic CH), 0.96 (methyl CH_3_) or 0.97 Å (methylene CH_2_). Isotropic displacement parameters for H atoms were fixed to *U*_iso_(H7x) = 1.5*U*_eq_(C7) and *U*_iso_(H) = 1.2*U*_eq_(carrier C) for other H atoms.

### Crystal data

**Table:**

C_16_H_14_Cl_2_O_4_S	*Z* = 2
*M**_r_* = 373.23	*F*(000) = 384
Triclinic, *P*1	*D*_x_ = 1.448 Mg m^−^^3^
Hall symbol: -P 1	Mo *K*α radiation, λ = 0.71073 Å
*a* = 8.8694 (8) Å	Cell parameters from 1336 reflections
*b* = 9.7501 (9) Å	θ = 2.8–23.7°
*c* = 10.3796 (11) Å	µ = 0.52 mm^−^^1^
α = 83.369 (2)°	*T* = 298 K
β = 76.196 (1)°	Prism, colourless
γ = 80.038 (1)°	0.45 × 0.40 × 0.30 mm
*V* = 855.95 (14) Å^3^	

### Data collection

**Table:**

Bruker SMART APEX CCD area-detector diffractometer	2967 independent reflections
Radiation source: fine-focus sealed tube	1762 reflections with *I* > 2σ(*I*)
graphite	*R*_int_ = 0.017
ω and φ scans	θ_max_ = 25.0°, θ_min_ = 2.8°
Absorption correction: multi-scan (*SADABS*; Sheldrick, 1996)	*h* = −10→9
*T*_min_ = 0.801, *T*_max_ = 0.860	*k* = −11→11
4290 measured reflections	*l* = −9→12

### Refinement

**Table:**

Refinement on *F*^2^	Primary atom site location: structure-invariant direct methods
Least-squares matrix: full	Secondary atom site location: difference Fourier map
*R*[*F*^2^ > 2σ(*F*^2^)] = 0.048	Hydrogen site location: inferred from neighbouring sites
*wR*(*F*^2^) = 0.140	H-atom parameters constrained
*S* = 1.02	*w* = 1/[σ^2^(*F*_o_^2^) + (0.0575*P*)^2^ + 0.4401*P*] where *P* = (*F*_o_^2^ + 2*F*_c_^2^)/3
2967 reflections	(Δ/σ)_max_ = 0.001
209 parameters	Δρ_max_ = 0.36 e Å^−^^3^
0 restraints	Δρ_min_ = −0.33 e Å^−^^3^
0 constraints	

### Fractional atomic coordinates and isotropic or equivalent isotropic displacement parameters (Å^2^)

**Table:**

	*x*	*y*	*z*	*U*_iso_*/*U*_eq_	
Cl1	0.82040 (13)	0.91886 (11)	1.44301 (11)	0.0879 (4)	
Cl2	0.75166 (16)	1.04135 (13)	1.72255 (11)	0.0992 (4)	
O1	0.5562 (2)	1.4568 (2)	1.2109 (2)	0.0595 (6)	
O2	0.4219 (3)	1.2645 (3)	1.1872 (3)	0.0871 (9)	
O3	0.2975 (3)	1.4507 (3)	1.3373 (3)	0.0915 (9)	
O4	0.8069 (3)	1.4105 (3)	1.3153 (2)	0.0630 (6)	
S1	0.43068 (11)	1.35611 (11)	1.28005 (11)	0.0703 (3)	
C1	0.7014 (4)	1.3996 (3)	1.1322 (3)	0.0552 (9)	
C2	0.8326 (4)	1.3787 (3)	1.1873 (3)	0.0548 (8)	
C3	0.9776 (4)	1.3308 (4)	1.1077 (4)	0.0653 (10)	
H3	1.0669	1.3146	1.1428	0.078*	
C4	0.9901 (5)	1.3068 (4)	0.9758 (4)	0.0733 (11)	
C5	0.8575 (6)	1.3299 (4)	0.9242 (4)	0.0807 (12)	
H5	0.8659	1.3141	0.8359	0.097*	
C6	0.7135 (5)	1.3759 (4)	1.0020 (4)	0.0716 (11)	
H6	0.6244	1.3909	0.9669	0.086*	
C7	0.9350 (5)	1.3767 (6)	1.3797 (4)	0.0961 (15)	
H7A	0.9763	1.2793	1.3742	0.144*	
H7B	0.8993	1.3964	1.4715	0.144*	
H7C	1.0157	1.4315	1.3369	0.144*	
C8	1.1522 (6)	1.2558 (5)	0.8904 (4)	0.0989 (16)	
H8A	1.1523	1.2820	0.7973	0.119*	
H8B	1.2304	1.3008	0.9136	0.119*	
C9	1.1938 (7)	1.1033 (5)	0.9099 (4)	0.1003 (16)	
H9	1.1251	1.0486	0.8940	0.120*	
C10	1.3139 (8)	1.0416 (7)	0.9462 (5)	0.133 (2)	
H10A	1.3855	1.0926	0.9632	0.159*	
H10B	1.3318	0.9445	0.9563	0.159*	
C11	0.5144 (4)	1.2605 (4)	1.4070 (3)	0.0578 (9)	
C12	0.6161 (4)	1.1383 (4)	1.3789 (3)	0.0564 (9)	
H12	0.6347	1.1026	1.2964	0.068*	
C13	0.6895 (4)	1.0704 (4)	1.4771 (4)	0.0588 (9)	
C14	0.6590 (4)	1.1249 (4)	1.6005 (4)	0.0651 (10)	
C15	0.5542 (5)	1.2442 (4)	1.6267 (4)	0.0728 (11)	
H15	0.5319	1.2779	1.7105	0.087*	
C16	0.4817 (4)	1.3143 (4)	1.5303 (4)	0.0682 (10)	
H16	0.4120	1.3965	1.5473	0.082*	

### Atomic displacement parameters (Å^2^)

**Table:**

	*U*^11^	*U*^22^	*U*^33^	*U*^12^	*U*^13^	*U*^23^
Cl1	0.0900 (8)	0.0784 (7)	0.0890 (8)	0.0078 (6)	−0.0208 (6)	−0.0102 (6)
Cl2	0.1245 (11)	0.1074 (9)	0.0685 (7)	−0.0214 (8)	−0.0319 (7)	0.0095 (6)
O1	0.0484 (14)	0.0575 (14)	0.0743 (16)	0.0003 (11)	−0.0208 (12)	−0.0098 (12)
O2	0.089 (2)	0.091 (2)	0.099 (2)	−0.0254 (16)	−0.0462 (17)	−0.0127 (16)
O3	0.0432 (15)	0.109 (2)	0.120 (2)	0.0058 (15)	−0.0224 (15)	−0.0165 (18)
O4	0.0466 (13)	0.0839 (17)	0.0603 (15)	−0.0047 (12)	−0.0146 (11)	−0.0154 (13)
S1	0.0509 (6)	0.0797 (7)	0.0867 (7)	−0.0086 (5)	−0.0258 (5)	−0.0125 (5)
C1	0.057 (2)	0.049 (2)	0.060 (2)	−0.0025 (16)	−0.0165 (17)	−0.0059 (16)
C2	0.054 (2)	0.049 (2)	0.060 (2)	−0.0053 (16)	−0.0118 (17)	−0.0059 (16)
C3	0.058 (2)	0.061 (2)	0.068 (2)	−0.0009 (18)	−0.0059 (19)	0.0014 (18)
C4	0.086 (3)	0.053 (2)	0.063 (3)	0.005 (2)	0.004 (2)	0.0011 (19)
C5	0.103 (4)	0.074 (3)	0.059 (2)	−0.001 (3)	−0.015 (2)	−0.008 (2)
C6	0.084 (3)	0.068 (3)	0.064 (2)	0.000 (2)	−0.026 (2)	−0.005 (2)
C7	0.057 (2)	0.158 (5)	0.082 (3)	−0.004 (3)	−0.034 (2)	−0.023 (3)
C8	0.101 (3)	0.076 (3)	0.086 (3)	0.011 (3)	0.023 (3)	0.002 (2)
C9	0.109 (4)	0.095 (4)	0.073 (3)	0.018 (3)	0.008 (3)	−0.017 (3)
C10	0.156 (6)	0.141 (5)	0.078 (3)	0.031 (5)	−0.014 (4)	−0.017 (3)
C11	0.048 (2)	0.062 (2)	0.066 (2)	−0.0153 (17)	−0.0104 (17)	−0.0087 (18)
C12	0.051 (2)	0.062 (2)	0.059 (2)	−0.0159 (18)	−0.0095 (17)	−0.0103 (17)
C13	0.054 (2)	0.057 (2)	0.065 (2)	−0.0165 (17)	−0.0068 (18)	−0.0049 (18)
C14	0.070 (2)	0.072 (3)	0.055 (2)	−0.026 (2)	−0.0103 (18)	0.0020 (19)
C15	0.081 (3)	0.076 (3)	0.059 (2)	−0.018 (2)	−0.002 (2)	−0.014 (2)
C16	0.062 (2)	0.066 (2)	0.071 (3)	−0.0100 (19)	0.001 (2)	−0.017 (2)

### Geometric parameters (Å, °)

**Table:**

Cl1—C13	1.729 (4)	C7—H7A	0.9600
Cl2—C14	1.724 (4)	C7—H7B	0.9600
O1—C1	1.411 (4)	C7—H7C	0.9600
O1—S1	1.598 (2)	C8—C9	1.471 (6)
O2—S1	1.411 (3)	C8—H8A	0.9700
O3—S1	1.419 (3)	C8—H8B	0.9700
O4—C2	1.356 (4)	C9—C10	1.247 (7)
O4—C7	1.424 (4)	C9—H9	0.9300
S1—C11	1.761 (4)	C10—H10A	0.9300
C1—C6	1.372 (5)	C10—H10B	0.9300
C1—C2	1.390 (4)	C11—C12	1.379 (4)
C2—C3	1.389 (5)	C11—C16	1.387 (5)
C3—C4	1.391 (5)	C12—C13	1.383 (5)
C3—H3	0.9300	C12—H12	0.9300
C4—C5	1.378 (6)	C13—C14	1.391 (5)
C4—C8	1.531 (5)	C14—C15	1.367 (5)
C5—C6	1.371 (6)	C15—C16	1.371 (5)
C5—H5	0.9300	C15—H15	0.9300
C6—H6	0.9300	C16—H16	0.9300
			
C1—O1—S1	119.2 (2)	H7B—C7—H7C	109.5
C2—O4—C7	117.7 (3)	C9—C8—C4	111.4 (4)
O2—S1—O3	121.23 (17)	C9—C8—H8A	109.4
O2—S1—O1	109.01 (16)	C4—C8—H8A	109.4
O3—S1—O1	102.96 (16)	C9—C8—H8B	109.4
O2—S1—C11	109.38 (17)	C4—C8—H8B	109.4
O3—S1—C11	109.49 (18)	H8A—C8—H8B	108.0
O1—S1—C11	103.13 (14)	C10—C9—C8	125.2 (6)
C6—C1—C2	121.5 (3)	C10—C9—H9	117.4
C6—C1—O1	120.5 (3)	C8—C9—H9	117.4
C2—C1—O1	117.9 (3)	C9—C10—H10A	120.0
O4—C2—C3	125.6 (3)	C9—C10—H10B	120.0
O4—C2—C1	116.1 (3)	H10A—C10—H10B	120.0
C3—C2—C1	118.3 (3)	C12—C11—C16	122.1 (3)
C2—C3—C4	120.3 (4)	C12—C11—S1	119.1 (3)
C2—C3—H3	119.8	C16—C11—S1	118.7 (3)
C4—C3—H3	119.8	C11—C12—C13	118.1 (3)
C5—C4—C3	119.7 (4)	C11—C12—H12	121.0
C5—C4—C8	121.3 (4)	C13—C12—H12	121.0
C3—C4—C8	118.9 (4)	C12—C13—C14	120.1 (3)
C6—C5—C4	120.5 (4)	C12—C13—Cl1	118.9 (3)
C6—C5—H5	119.7	C14—C13—Cl1	121.0 (3)
C4—C5—H5	119.7	C15—C14—C13	120.5 (3)
C5—C6—C1	119.7 (4)	C15—C14—Cl2	119.3 (3)
C5—C6—H6	120.2	C13—C14—Cl2	120.2 (3)
C1—C6—H6	120.2	C14—C15—C16	120.4 (4)
O4—C7—H7A	109.5	C14—C15—H15	119.8
O4—C7—H7B	109.5	C16—C15—H15	119.8
H7A—C7—H7B	109.5	C15—C16—C11	118.8 (4)
O4—C7—H7C	109.5	C15—C16—H16	120.6
H7A—C7—H7C	109.5	C11—C16—H16	120.6
			
C1—O1—S1—O2	−44.3 (3)	C3—C4—C8—C9	83.8 (5)
C1—O1—S1—O3	−174.3 (2)	C4—C8—C9—C10	−123.3 (6)
C1—O1—S1—C11	71.8 (3)	O2—S1—C11—C12	27.3 (3)
S1—O1—C1—C6	82.4 (4)	O3—S1—C11—C12	162.3 (3)
S1—O1—C1—C2	−102.5 (3)	O1—S1—C11—C12	−88.6 (3)
C7—O4—C2—C3	−8.0 (5)	O2—S1—C11—C16	−155.9 (3)
C7—O4—C2—C1	173.5 (3)	O3—S1—C11—C16	−20.9 (3)
C6—C1—C2—O4	177.7 (3)	O1—S1—C11—C16	88.2 (3)
O1—C1—C2—O4	2.6 (5)	C16—C11—C12—C13	−1.5 (5)
C6—C1—C2—C3	−1.0 (5)	S1—C11—C12—C13	175.2 (2)
O1—C1—C2—C3	−176.0 (3)	C11—C12—C13—C14	0.6 (5)
O4—C2—C3—C4	−177.4 (3)	C11—C12—C13—Cl1	−178.8 (3)
C1—C2—C3—C4	1.0 (5)	C12—C13—C14—C15	1.2 (5)
C2—C3—C4—C5	−0.5 (6)	Cl1—C13—C14—C15	−179.4 (3)
C2—C3—C4—C8	179.2 (3)	C12—C13—C14—Cl2	−179.4 (3)
C3—C4—C5—C6	−0.2 (6)	Cl1—C13—C14—Cl2	−0.1 (4)
C8—C4—C5—C6	−179.9 (4)	C13—C14—C15—C16	−2.2 (6)
C4—C5—C6—C1	0.3 (6)	Cl2—C14—C15—C16	178.5 (3)
C2—C1—C6—C5	0.3 (6)	C14—C15—C16—C11	1.3 (6)
O1—C1—C6—C5	175.2 (3)	C12—C11—C16—C15	0.6 (5)
C5—C4—C8—C9	−96.5 (5)	S1—C11—C16—C15	−176.2 (3)
